# Clinical use of rituximab in haematological malignancies

**DOI:** 10.1038/sj.bjc.6601187

**Published:** 2003-10-14

**Authors:** I Avivi, S Robinson, A Goldstone

**Affiliations:** 1 University College London Hospital, UK; 2 Bristol Royal Hospital for Children, UK

**Keywords:** rituximab, haematological, malignancies

## Abstract

Rituximab is a chimeric human/mouse monoclonal antibody that is approved for the treatment of relapsed and refractory non-Hodgkin's lymphoma (NHL) and in combination with CHOP (cyclophosphamide, doxorubicin, vincristine, prednisone) chemotherapy as first-line therapy for diffuse large B-cell NHL, where it has shown the first survival advantage over CHOP alone in more than 20 years. Strategies to help define the optimal therapeutic usage of rituximab are being assessed, including first-line and maintenance or extended therapy, and the combination of rituximab with chemotherapy in indolent NHL. Emerging data suggest that earlier use may yield higher response rates, extended therapy can prolong remission, and the addition of rituximab to chemotherapy can increase clinical and molecular remission rates when compared with those achieved using chemotherapy alone. Studies in the peritransplant setting suggest a role for rituximab *in vivo* purging prior to transplant and/or maintenance rituximab as a means of clearing minimal residual disease. Rituximab has also shown activity in other B-cell disorders such as chronic lymphocytic leukaemia. The full potential of this immunotherapeutic agent remains to be defined in ongoing and future clinical trials.

Combination chemotherapy has markedly improved outcome in aggressive non-Hodgkin's lymphoma (NHL) since its introduction in the 1970s, but is curative in less than half of the patients. In indolent NHL, the impact of chemotherapy has been much lower; while response rates and progression-free survival (PFS) may be improved, overall survival (OS) has not increased and advanced indolent NHL remains essentially incurable without allogeneic bone marrow transplant.

A number of newer approaches to improve outcome are under investigation, including the use of monoclonal antibodies targeted against specific antigens expressed by lymphoma cells. The cell surface antigen CD20 is in many ways an ideal target: it is expressed by 95% of B-cell NHL cases but not on haematopoietic stem cells; it has a functional role in B-cell growth and also does not normally circulate as a free antigen in the plasma, so free antigen does not compete for antibody binding.

Rituximab is an anti-CD20 chimeric monoclonal antibody and was the first antibody approved for treatment of NHL. Multiple mechanisms of action for rituximab have been identified by *in vitro* studies. These include complement-dependent cytotoxicity (CDC), antibody-dependent cellular cytotoxicity (ADCC) and induction of apoptosis (reviewed by Maloney *et al*, 2002). Rituximab is also able to sensitise lymphoma cells to the cytotoxic activity of chemotherapy. There is now a substantial body of data describing the clinical use of rituximab in NHL, which we review here.

## PREVIOUSLY UNTREATED INDOLENT NHL

Several studies have examined rituximab as a first-line therapy in patients with indolent NHL (Colombat *et al*, 2001; Witzig *et al*, 2002; Hainsworth, 2003). This treatment approach has yielded consistently high response rates (overall response rate (ORR) 61–73%, with 20–37% complete responses (CR)) (Colombat *et al*, 2001; Witzig *et al*, 2002; Hainsworth, 2003) (Table 1

**Table 1:** Rituximab in NHL

**Disease setting**	**Therapy**	***n***	**ORR (%)**	**CR (%)**	**Reference**
Untreated indolent	Single-agent rituximab	50	73	27	Colombat *et al* (2001)
Untreated indolent	Single-agent rituximab	60	47	7	Hainsworth (2003)
Untreated indolent	Single-agent rituximab	37	62	44	Witzig *et al* (2002)
Relapsed aggressive	Rituximab+EPOCH	50	64	26	Jost *et al* (2001)
Relapsed aggressive	Rituximab+ICE	31	81	55	Kewalramani *et al* (2001)
Relapsed aggressive	Rituximab+paclitaxel/ topotecan	45	69	44	Younes *et al* (2001)
Untreated CLL	Rituximab+fludarabine	104	90	47	Byrd *et al* (2003)
Untreated/relapsed CLL	Rituximab+fludarabine	30	90	33	Schulz *et al* (2002)
Untreated CLL	Rituximab+fludarabine/ cyclophosphamide	135	95	67	Wierda *et al* (2002)

). It appears that extended therapy (induction followed by maintenance) may be better than rituximab induction only: recent 2-year follow-up data from a phase II trial of first-line and maintenance rituximab (Hainsworth, 2003) showed high ORRs and CRs (73 and 37%, respectively), and a median PFS of 34 months at a median 30-month follow-up. A phase III trial has compared maintenance therapy *vs* observation only following rituximab induction (Ghielmini *et al*, 2002). Of 202 patients enrolled, 151 responded or had stable disease following a 4-week course of rituximab, and were randomised to either rituximab maintenance (four once-weekly doses, every 2 months × 4) or to observation. At a median follow-up of 35 months, the rituximab maintenance group was found to have a 43% reduced risk of progression (median event-free survival (EFS) 23 *vs* 12 months; *P*=0.02), with no increase in treatment-related adverse events. In the previously untreated patients, the difference in EFS was even greater (36 *vs* 19 months; *P*=0.009).

The efficacy and tolerability of rituximab suggest that it may be particularly useful as a first-line single-agent therapy in elderly patients, and also in young women who want to preserve their fertility. It may also have a role as a first-line therapy in asymptomatic patients with advanced disease (stages 3 or 4), aiming to prolong the asymptomatic period. This will need to be assessed in clinical trials, however.

### Rituximab plus chemotherapy in previously untreated indolent NHL

The single-agent activity of rituximab, coupled with its distinct mechanisms of action, nonoverlapping toxicity and ability to sensitise lymphoma cells to cytotoxic activity, has encouraged researchers to evaluate combinations of rituximab with chemotherapy. In a phase II trial, rituximab plus chemotherapy (CHOP or CVP) has demonstrated high activity, achieving a response rate of around 97% (57% CR) in 82 patients with previously untreated follicular lymphoma (Hainsworth *et al*, 2002). In all, 85% of patients with follicular lymphoma carry a characteristic chromosomal translocation, t(14;18), leading to overexpression of *bcl-2*, an antiapoptotic gene that may serve as a survival factor for lymphoma cells and can be detected by polymerase chain reaction (PCR). Achievement of molecular remission post-therapy (clearance of *bcl-2* positive cells) appears to be associated with prolonged PFS and may even predict longer OS (Freedman *et al*, 1999).

Another study with rituximab plus FND (fludarabine, novantrone, dexamethasone) chemotherapy has shown high rates of molecular remission in the peripheral blood (90%) and bone marrow (76%) of patients with previously untreated follicular lymphoma at a 6-month follow-up (Cabanillas *et al*, 2000). The most extensively evaluated regimen, rituximab plus CHOP, has produced response rates of up to 100% (including 58% CRs) in both treatment-naïve and pretreated follicular lymphoma patients, with an impressive median PFS in excess of 65 months (Czuczman *et al*, 2001b). Similar results have been obtained using rituximab plus fludarabine (Czuczman *et al*, 2001a). Rituximab-containing chemotherapy regimens appear to increase both clinical and molecular remission rates when compared with those usually achieved with chemotherapy only, giving grounds for optimism that they may also result in improved PFS.

Rituximab has also been assessed as consolidation treatment in patients with minimal residual disease (MRD) (*bcl-*2 positivity in the presence of complete clinical remission) after CHOP or fludarabine/mitoxantrone (FM) chemotherapy (Zinzani, 2002). The aim of the consolidation treatment was to obtain molecular remission and prolong PFS. An increase in molecular remission was observed after rituximab consolidation in both chemotherapy groups, from 20 to 40% in the CHOP group and from 34 to 59% in the FM group. Sequential therapy with short-course chemotherapy followed by rituximab has also been investigated in previously untreated patients (Rambaldi *et al*, 2002), and the results are similar to those for concurrent therapy. The combination of rituximab plus CVP (chlorambucil, vincristine, prednisone) *vs* CVP alone as first-line therapy for indolent NHL is currently being studied in a phase III randomised trial (M39021 Trial).

## RELAPSED INDOLENT NHL

Rituximab is established in the treatment of relapsed indolent NHL, being both effective and well tolerated. The pivotal trial in this setting included 166 patients with relapsed low-grade or follicular lymphoma who were assigned to receive four once-weekly doses of rituximab 375 mg m^−2^ (McLaughlin *et al*, 1998). The ORR was 48%, with a median time to progression (TTP) of 13 months in responders.

Prolonged single-agent rituximab (Piro *et al*, 1999) and/or retreatment (Davis *et al*, 2000) in patients with relapsed disease are both feasible and may have a beneficial effect on response rate and PFS. Extended rituximab therapy has been shown to achieve a response rate of 60% and a median disease-free survival in excess of 19 months (Piro *et al*, 1999), providing a rationale for maintenance rituximab in the first-line setting. These strategies may be particularly useful for patient groups such as the elderly, who may not be able to tolerate conventional chemotherapy or immunochemotherapy.

### Rituximab plus chemotherapy for relapsed indolent NHL

Single-agent rituximab is active in patients with relapsed indolent lymphoma, but the remissions are no more durable than with conventional chemotherapy, and patients eventually relapse. Attempts to improve PFS by combining rituximab with chemotherapy are currently being undertaken. A study by the German Low-Grade Lymphoma Study Group is comparing the combination of fludarabine, cyclophosphamide and mitoxantrone (FCM) with rituximab plus FCM in patients with relapsed follicular and mantle cell lymphoma (MCL), and interim analyses have reported a response rate of 83% for the immunochemotherapy arm compared with 53% for FCM alone (Hiddemann *et al*, 2001), indicating that combined modality treatment may be superior to chemotherapy alone.

Many studies now suggest that combination immunochemotherapy may be more effective than single-agent rituximab or chemotherapy alone, and thus this treatment may be offered to patients with relapsed disease who can tolerate a combined regimen. However, the results of prospective comparative trials are still needed to confirm the superiority of immunochemotherapy in this setting.

## MANTLE CELL LYMPHOMA

Patients with MCL have poor prognosis and curative treatment options are limited. As the majority of MCL express CD20, there is a clear rationale for the use of rituximab in this setting. A large, phase II study, assessing the efficacy of single-agent rituximab in 74 patients with newly diagnosed or relapsed MCL (34 and 40 patients, respectively), demonstrated a response rate of 34% with a median PFS of 14 months (Foran *et al*, 2000), confirming the activity of rituximab in MCL.

Given the poor prognosis of MCL, high-dose therapy (HDT) with autologous stem cell transplantation (ASCT) is being increasingly considered for patients achieving their first complete remission. Addition of rituximab to HDT/ASCT has been evaluated by Mangel *et al* (2002), who, in a matched pair analysis, showed that patients who received rituximab purging during intensive conditioning for ASCT achieved significantly better PFS, with a trend towards longer OS, than historical controls who received conventional chemotherapy (anthracycline or cyclophosphamide/fludarabine). Another study in this setting found that all 20 assessable patients achieved lymphoma-free stem cell harvests (by PCR analysis) following intensified induction with rituximab *in vivo* purging, while 26 of the 28 patients were alive and disease-free at a median follow-up of 22 months (Gianni *et al*, 2002). Romaguera *et al* (2001) treated 77 patients with previously untreated MCL with a combination of rituximab plus HyperCVAD. A response rate of 89% was obtained and, interestingly, failure-free survival and OS in younger patients were similar to that previously achieved using the HyperCVAD regimen in combination with HDT/ASCT. The addition of rituximab to the HyperCVAD regimen may, therefore, result in durable remissions without the need for ASCT.

## RITUXIMAB IN AGGRESSIVE NHL

The activity of single-agent rituximab in relapsed aggressive NHL has been demonstrated, but the strongest data in aggressive NHL have come from studies of combination immunochemotherapy, particularly rituximab plus CHOP (R-CHOP). Vose *et al* (2001) reported a response rate of 94%, with 61% CR, for R-CHOP in 33 patients with previously untreated aggressive NHL. Long-term (median 62-month) follow-up of these 33 patients has recently reported an OS and PFS of 87 and 80%, respectively (Vose *et al*, 2002).

A prospective randomised trial in 399 elderly patients (aged 60–80 years) with previously untreated diffuse large B-cell lymphoma (DLCL) demonstrated a significant advantage for R-CHOP over CHOP alone (Coiffier *et al*, 2002). Patients in the R-CHOP group had a higher CR rate (75 *vs* 63%, *P*=0.005), improved PFS (2-year disease progression 9 *vs* 22%, *P*=0.007; 2-year relapse rate 14 *vs* 25%, *P*=0.002) and increased EFS and OS (2-year EFS 57 *vs* 38%, *P*<0.001; 2-year OS 70 *vs* 57%, *P*=0.007). No significant difference in treatment-related toxicity was seen between the groups. In an interim subset analysis of 328 patients, it was shown that R-CHOP was superior to CHOP alone in both low-risk and high-risk patients, and also in patients aged 60–70 or 70–80 years (Coiffier *et al*, 2001). This study has identified R-CHOP as the gold standard therapy in elderly patients with DLCL and the results of a similar ongoing study in younger patients are awaited (MInT).

### Rituximab for relapsed aggressive NHL

HDT followed by ASCT is the preferred treatment for younger patients with relapsed aggressive lymphoma that is responsive to conventional salvage regimens. Several studies have shown good ORRs and CRs (ORR 60–80% with CR 25–60%) in patients treated with a rituximab-containing salvage regimen which appear superior to those achieved with chemotherapy alone (Table 1).

## RITUXIMAB IN STEM CELL TRANSPLANTATION

While HDT/ASCT may be considered a potentially curative therapy for aggressive NHL, approximately 40–60% of patients with aggressive NHL, and most patients with indolent NHL, will eventually relapse following transplant. Relapse post-transplant is due either to contamination of the stem cell harvest or persistence of MRD post-HDT. A number of strategies have therefore been employed in attempts to prevent/reduce stem cell contamination (stem cell purging) and/or eradicate lymphoma cells that survived HDT (post-transplant ‘mopping up’).

Patients with follicular lymphoma whose bone marrow harvests are cleared of *bcl-2*-positive cells have increased relapse-free survival compared with patients whose bone marrow harvests remain positive (Freedman *et al*, 1999). Whereas the efficacy of immunological *ex vivo* purging (using monoclonal antibodies) is limited (up to 58% of harvests remain *bcl*-2 positive), *in vivo* purging with rituximab has produced *bcl-*2-negative harvests in 69–90% of stem cell harvests without compromising stem cell yield or function (reviewed by Gisselbrecht and Mounier, 2003).

Rituximab *in vivo* purging pretransplant results in a high rate of molecular remissions post-transplant, and favourable PFS (reviewed by Gisselbrecht and Mounier, 2003). A number of studies have demonstrated an increase in molecular remission using rituximab post-transplant (Horwitz *et al*, 2001; Brugger *et al*, 2002), but it remains to be seen whether these translate into durable clinical remissions. Whether there is, in fact, a significant survival advantage using rituximab peritransplant, however, remains to be determined in prospective randomised studies. The ongoing EBMT Lym-1 trial randomises patients with follicular lymphoma to receive ASCT with or without *in vivo* purging with rituximab, as well as to post-transplant rituximab *vs* observation. This and other ongoing studies in both indolent and aggressive NHL will help to define the role of rituximab in the peritransplant setting.

## RITUXIMAB IN CHRONIC LYMPHOCYTIC LEUKAEMIA

The efficacy and tolerability of rituximab have been evaluated in other haematological disorders, most notably chronic lymphocytic leukaemia (CLL) and small lymphocytic lymphoma (SLL), the lymphomatous equivalent of CLL. However, single-agent rituximab has yielded low response rates (approximately 15%) in patients with relapsed CLL/SLL (McLaughlin *et al*, 1998; Piro *et al*, 1999). In contrast, the response rate in patients with previously untreated CLL/SLL was much higher (56%, with 8% CR) (Hainsworth, 2003). Given the low responses seen in relapsed disease, increased dosing and dose escalation have been evaluated as strategies to increase response to rituximab (Byrd *et al*, 2001; O'Brien *et al*, 2001). Both were shown to increase response rates to between 45 and 75%, but these strategies are not feasible in routine medical practice. The combination of chemotherapy with rituximab appears to be well tolerated and effective in CLL. Studies of rituximab with fludarabine-containing regimens, based on previous data showing an *in vitro* synergy between fludarabine and rituximab (Alas *et al*, 2000), reported high response rates of 90–95% (CR of 33–67%) (Schulz *et al*, 2002; Wierda *et al*, 2002; Byrd *et al*, 2003) (Table 1). The impact of these clinical responses on PFS and OS remains to be determined.

## RITUXIMAB IN OTHER HAEMATOLOGICAL DISORDERS

Rituximab has been evaluated in a range of other B-cell malignancies, including post-transplant lymphoproliferative disorder (PTLD), Waldenström's disease and HIV-associated lymphoma. Data from two studies indicate that approximately 50% of the patients with PTLD may respond to rituximab monotherapy, and CRs of 33 and 52% have been reported (Choquet *et al*, 2002; Oertel *et al*, 2002). Rituximab has produced response rates of 40–86% in Waldenström's macroglobulinaemia with some evidence for CRs, especially when used in combination with fludarabine (Byrd *et al*, 1999; Treon *et al*, 2002). Patients with HIV-related NHL have shown very encouraging responses to rituximab with ORRs and CR rates of approximately 80% (Boue *et al*, 2002). Although patient numbers are low, the data for rituximab in lymphocyte predominant Hodgkin's disease are also encouraging.

Rituximab has also been evaluated in nonmalignant B-cell disorders. In immune thrombocytopenic purpura (ITP), single-agent rituximab has demonstrated responses in up to 72% of patients, with CR in 32% and a response duration of 12+ months for patients in CR (Cooper *et al*, 2002). The highest response rates have been seen in patients with refractory ITP (Cooper *et al*, 2002), suggesting that rituximab may be a valuable agent for patients with few other treatment options. Promising results have also been demonstrated in autoimmune haemolytic anaemia, where all patients have responded to both treatment and retreatment with rituximab (Gupta *et al*, 2001). Taken together, the studies indicate the potential of rituximab in B-cell disorders, and the need for further assessment of its efficacy and tolerability in these settings.

## TOLERABILITY OF RITUXIMAB

Single-agent studies have reported the good tolerability of rituximab. The most common adverse events are infusion-related reactions, comprising mainly fever and chills, in more than 50% of patients. These occur usually within hours of the first infusion and are rare with subsequent infusions. No significant increase in infusion-related reactions is seen in patients treated with rituximab plus chemotherapy. In the phase II trial of rituximab plus CHOP *vs* CHOP alone, grade 3 or 4 infusion-related reactions were seen in 9% of patients in the rituximab plus CHOP arm, but all were able to complete planned therapy with no further recurrence of severe infusion-related reactions (Coiffier *et al*, 2002). Subgroups of patients who may be at higher risk of rituximab-associated adverse events have been identified. Patients with high tumour burden are at increased risk of tumour lysis syndrome. Those with existing or prior pulmonary or cardiac complications should also be treated with caution and monitored because of infusion-related reactions.

Single-agent rituximab induces B-cell depletion in the majority of patients (McLaughlin *et al*, 1998). Given this B-cell depletion, monitoring of infections has been undertaken. During treatment in the McLaughlin trial (single-agent rituximab in relapsed low-grade and follicular NHL), 50 of 166 patients developed 68 infectious events (McLaughlin *et al*, 1998). Of these, six events (only 9%) were grade 3 and none grade 4. Prolonged B-cell depletion with maintenance rituximab also does not seem to result in a significant increase in infections (Ghielmini *et al*, 2002; Hainsworth, 2003). Furthermore, combination studies indicate that the addition of rituximab to chemotherapy does not significantly increase clinically relevant toxicity. In the phase III study of rituximab plus CHOP *vs* CHOP (Coiffier *et al*, 2002), the incidence of grade 3 or 4 adverse events was very similar in both arms.

Data on the development of human anti-mouse (HAMA) or human anti-chimeric antibody (HACA) are available (Rituximab Summary of Product Characteristics). Of 67 patients evaluated for HAMA, no responses were observed; of 356 evaluated for HACA, 1.1% (four patients) were positive. These data indicate that retreatment or maintenance therapy with rituximab remains feasible: patients may continue responding to repeated rituximab exposure without developing immune resistance.

## FUTURE DIRECTIONS

Several trials are ongoing or planned in order to better define the role of rituximab in therapy for indolent (Table 2

**Table 2:** Ongoing studies of rituximab in indolent NHL

**Group/study**	***n***	**Treatment**
Nordic lymphoma group	318	Rituximab induction±interferon in untreated indolent NHL. Patients with partial or minor response receive further rituximab induction±interferon
ECOG 1496	515	Rituximab maintenance after CVP in untreated indolent lymphoma
EORTC-20981	600	CHOP±rituximab±maintenance treatment in relapsed follicular NHL
EBMT Lym-1	460	Rituximab purging and maintenance therapy with ASCT in relapsed follicular NHL
M39021	322	CVP±rituximab in untreated indolent lymphoma

) and aggressive NHL (Table 3

**Table 3:** Ongoing studies of rituximab immunochemotherapy in aggressive NHL

**Group/study**	***n***	**Treatment**
ECOG 4494	631	CHOP±rituximab±maintenance in older patients with diffuse large cell NHL
MInt	820	CHOP±rituximab in younger untreated patients with DLCL
AMC-010		CHOP±rituximab in untreated HIV-associated NHL
RICOVER 60	880	2 × 2 factorial design: CHOP 14±rituximab and six *vs* eight cycles of chemotherapy (eight doses rituximab) in older patients with untreated aggressive NHL
CORAL trial	400	Rituximab+ICE *vs* rituximab+DHAP in relapsed aggressive NHL followed by HDT/ASCT. Second randomisation to rituximab maintenance *vs* observation

) and for other B-cell disorders. In the indolent setting there is a growing focus on first-line evaluation, combination therapy with either chemotherapy or other biological agents, and rituximab maintenance therapy. Of note is the EBMT Lym-1 trial assessing rituximab in the peritransplant setting both as an *in vivo* purge and as maintenance therapy. A BNLI trial of rituximab *vs* watchful waiting in patients with advanced-stage asymptomatic indolent NHL is planned.

In aggressive NHL, data from two ongoing trials are awaited with interest to confirm the clinical benefit of adding rituximab to CHOP (or CHOP-like therapy) for elderly patients (ECOG 4494) or younger patients (MInt) with untreated DLCL (Table 3). Several smaller phase II studies are evaluating the efficacy of rituximab in other B-cell disorders, one of which, central nervous system lymphomas, presents a particular challenge.

In summary, rituximab has shown efficacy and tolerability as monotherapy in indolent NHL. The data suggest that patients treated earlier in the course of their disease may respond better to therapy and that maintenance therapy may provide additional benefit. In both indolent and aggressive NHL, the use of rituximab with chemotherapy may provide an advantage over chemotherapy alone and this is reflected in the number of current and planned studies of immunochemotherapy. The survival benefit for rituximab plus CHOP over CHOP alone demonstrated in the GELA study is awaiting full confirmation by the MInT and ECOG trials in elderly and younger patients with untreated DLCL. The general trend seen in NHL is for improved response rates and quality of response when rituximab is added to chemotherapy: whether this will translate into improved survival will be determined by longer-term follow-up of completed studies. Transplantation studies indicate that rituximab *in vivo* purging is active and does not compromise ASCT although the role of both *in vivo* purging and post-transplant maintenance remains to be defined. One characteristic central to the integration of rituximab in these diverse clinical settings is its ability to achieve not only clinical but molecular responses–the mechanisms of action of this monoclonal antibody underpin its potential and utility in the treatment of CD20-positive disorders. An improved understanding of these mechanisms and the molecular basis of NHL will help the full therapeutic potential of this agent to be realised.
